# Maternal post-traumatic stress and depression symptoms and outcomes after NICU discharge in a low-income sample: a cross-sectional study

**DOI:** 10.1186/s12884-020-03536-0

**Published:** 2021-01-12

**Authors:** Kameelah Gateau, Ashley Song, Douglas L. Vanderbilt, Cynthia Gong, Philippe Friedlich, Michele Kipke, Ashwini Lakshmanan

**Affiliations:** 1 Division of Neonatology, LAC+USC Medical Center, Keck School of Medicine, University of Southern California, Los Angeles, CA USA; 2 Fetal and Neonatal Medicine Institute, Division of Neonatal Medicine, Children’s Hospital Los Angeles, Keck School of Medicine, University of Southern California, 4650 Sunset Boulevard, MS #31, Los Angeles, CA 90027 USA; 3 Department of Preventive Medicine, Johns Hopkins University, Baltimore, MD USA; 4 Section of Developmental-Behavioral Pediatrics, Children’s Hospital Los Angeles, Keck School of Medicine, University of Southern California, Los Angeles, CA USA; 5 Leonard D. Schaeffer Center for Health Policy and Economics, University of Southern California, Los Angeles, CA USA; 6 Division of Research on Children, Youth and Families, Children’s Hospital, Keck School of Medicine, University of Southern California, Los Angeles, CA USA; 7 Department of Preventive Medicine, Keck School of Medicine, University of Southern California, Los Angeles, CA USA

**Keywords:** Post-traumatic stress, Post partum depression, Low-income, NICU

## Abstract

**Background:**

Having a preterm newborn and the experience of staying in the neonatal intensive care unit (NICU) has the potential to impact a mother’s mental health and overall quality of life. However, currently there are few studies that have examined the association of acute post-traumatic stress (PTS) and depression symptoms and infant and maternal outcomes in low-income populations.

**Design/ methods:**

In a cross-sectional study, we examined adjusted associations between positive screens for PTS and depression using the Perinatal Post-traumatic stress Questionnaire (PPQ) and the Patient Health-Questionnaire 2 (PHQ-2) with outcomes using unconditional logistic and linear regression models.

**Results:**

One hundred sixty-nine parents answered the questionnaire with 150 complete responses. The majority of our sample was Hispanic (68%), non-English speaking (67%) and reported an annual income of <$20,000 (58%). 33% of the participants had a positive PPQ screen and 34% a positive PHQ-2 screen. After adjusting for confounders, we identified that a positive PHQ-2 depression score was associated with a negative unit (95% CI) change on the infant’s Vineland Adaptive Behavior Scales, second edition of − 9.08 (− 15.6, − 2.6) (*p* < 0.01). There were no significant associations between maternal stress and depression scores and infant Bayley Scales of Infant Development III scores or re-hospitalizations or emergency room visits. However, positive PPQ and screening score were associated with a negative unit (95% CI) unit change on the maternal Multicultural Quality of Life Index score of − 8.1 (− 12, − 3.9)(*p* < 0.01) and − 7.7 (− 12, − 3) (*p* = 0.01) respectively.

**Conclusions:**

More than one-third of the mothers in this sample screened positively for PTS and depression symptoms. Screening scores positive for stress and depression symptoms were associated with a negative change in some infant development scores and maternal quality of life scores. Thoughtful screening programs for maternal stress and depression symptoms should be instituted.

**Supplementary Information:**

The online version contains supplementary material available at 10.1186/s12884-020-03536-0.

## Background

Preterm birth is a significant contributor to neonatal and under five morbidity and mortality worldwide [1]. Of those who survive beyond the neonatal period, many very low birth weight (VLBW infants < 1500 g) [2] face significant lifelong disabilities including neurocognitive deficits and visual impairment, along with systemic illnesses including respiratory and cardiac disabilities while late preterm infants also have been shown to have poorer neurodevelopmental outcomes and worse Total School Readiness Scores at kindergarten [3, 4]. In addition to the morbidities preterm infants potentially face, there is also significant economic, psychosocial and emotional impact on the families [5].

Many studies have evaluated the various emotional and mental health challenges that the mothers of neonates, both term and preterm, that can present at birth and in the first few years after NICU discharge [6, 7]. One systematic review by Gavin et al. reviewed current available literature on the incidence and prevalence of perinatal depression, and found that in the time period during pregnancy to 3 months post-delivery, up to 19% of women have depressive symptoms with 7.1% of mothers having major depressive episodes [8]. Of the studies that have assessed mental health outcomes in mothers of preterm neonates, most studies describe mental health perturbations like post-traumatic stress disorder (PTSD) and depression as being strongly associated with the birth of preterm infants [8–15]. Prevalence rates for depression among mothers caring for preterm infants discharged from the NICU have been described to range between 28 and 40% [16]. Additionally, it has previously been established that when mothers develop mental health problems there is significant impact on parent-child attachment, cognitive, developmental and overall health outcomes in infants [17, 18]. Given that preterm neonates are a particularly vulnerable population already with an increased risk of poor health outcomes, it becomes of the utmost importance to identify the factors that contribute to the development of poor mental health outcomes amongst their mothers.

While many studies have evaluated some of the risk factors associated with developing poor mental health outcomes in mothers after their children have been discharged from the NICU, most of these studies have been in homogenous populations, and have not assessed parental maternal health in the discharge period, or have assessed only a few predictors of stress. Furthermore there are still very few studies that have been done amongst socioeconomically diverse populations that have also looked specifically at socio-demographic and medical outcomes of preterm infants post NICU discharge and how those factors potentially impact a mother’s overall well-being and mental health. In this study, our objectives were to 1) describe the prevalence of positive screens for acute posttraumatic stress and depression symptoms among low-income families after NICU discharge, 2) examine the adjusted association of PTS and depression symptoms and child neurodevelopmental and medical outcomes and 3) evaluate the adjusted association of PTS and depression symptoms and maternal quality of life.

## Methods

### Study design and participants

The study design was a single-center, cross-sectional study. One caregiver of preterm (< 37 weeks’ gestation) infants up to 24 months corrected age with completed developmental assessments attending a high-risk infant follow-up clinic at a quaternary urban children’s hospital between 2013 and 2015 was enrolled. A 150-item questionnaire with components validated in English and Spanish was administered to participants about life after discharge from the NICU.. Patient recruitment, survey administration and population characteristics are detailed in previous work [19, 20]. The Institutional Review Board at Children’s Hospital Los Angeles approved the study protocol. Written informed consent was obtained from all study participants.

### Measurements

A summary of the primary outcomes and mental health assessments along with developmental and adaptive assessments are listed below.

### Mental health

PTS and depression symptoms were assessed utilizing the modified Perinatal Posttraumatic Stress Disorder Questionnaire (PPQ) and the Patient Health Questionnaire 2 (PHQ-2). The original PPQ was designed to identify mothers who were experiencing symptoms of post-traumatic stress [21]. Through a series of 14 questions, mothers are asked using a 5-point scale (0 being not at all and 4 being often) to assess how frequently they experienced symptoms of post-traumatic stress including flashbacks, fear, avoidance, and hyper vigilance. The standard PPQ assesses these symptoms in individuals by administering it to participants who gave birth up to 4 months before the questionnaire was administered, and for that reason the modified PPQ was developed, which changed the phrasing to present tense to assess symptoms within the immediate postpartum period. The modified PPQ was utilized in our study [22]. Additionally, a PPQ screen was considered positive when a participant acknowledged the presence of six or more symptoms [7]. The PHQ-2 consists of 2 questions that assess the frequency of depression and anhedonia over the previous 2 weeks, with a score ranging from 0 to 6 [23]. The authors identify a cut-off score of 3 as a clinically meaningful value to screen for depression/anxiety [24]. These tools have been used in similar populations [21, 23].

### Medical outcome assessment

We asked parents questions about their infant’s health status since discharge including the number of emergency department visits, monthly clinic appointments and hospitalizations, immunizations, dependence on durable medical equipment, and administration of prescription medications.

### Neuro-developmental assessments

Early development was assessed using the Bayley Scales of Infant and Toddler Development, Third Edition (Bayley-III). The Bayley Scales identify children with developmental delay and assist with planning of appropriate interventions in children aged 1–42 months. The five distinct scales of assessment include Cognitive (91 items), Language (97 items), Motor (138 items), caregiver ratings of Social Emotional (35 items), and Adaptive Behavior (241 items). Cognition is subdivided into two categories: expressive language (48 items) and receptive language (49 items), and motor is assessed as both fine (66 items) and gross (72 items) tasks. The age-corrected mean score of the Bayley-III is 100 with a standard deviation of 15. A higher score indicates more advanced development [25].

### Vineland adaptive behavior scale II (VABS II)

Personal, social and communication skills were assessed using the Vineland Adaptive Behavior Scales, second edition (VABS-II) [26]. Adaptive behavior refers to an individual’s day-to-day activities needed for personal and social sustenance; these scales assess what a person does as opposed to what he or she should be able to do. There are four domains assessed: communication, daily living skills, socialization and motor skills. A composite score is provided across the four domains to summarize an individual’s performance. The age-corrected mean is 100 with a standard deviation of 15 with higher scores indicating higher function.

### Maternal quality of life

The multicultural quality of life index (MQLI) from Mezzich et al. was used to assess parent’s health status [27]. The MQLI was developed to assess health-related quality of life through targeted areas including: physical well-being, psychological/emotional well-being, self-care, occupational functioning, interpersonal functioning, social-emotional functioning, social emotional support, community and services support, personal and spiritual fulfillment. Participants were asked to rate these domains on a 10 point scale, with 1 being poor and 10 being excellent. They were also asked to self-report their overall health status through a series of 12 yes/no questions and a 5-point overall health rating scale ranging from poor to excellent. Questions asked included those assessing overall health status, limitation on physical activity, energy levels, depression, and pain.

### Statistical analysis

The characteristics of the study population were described using means and proportions. The frequency of covariates (race/ethnicity, income level, maternal education, language, infant birth weight, infant gestational age, neonatal co-morbidities, use of medical equipment and post discharge diagnoses) were compared across PPQ and PHQ-2 scores. *P*-values were derived using t-tests for two group comparisons. Given the small subset of fathers, fathers’ responses were excluded from the multivariable analysis. Multivariable logistic regression estimated the adjusted odds of readmissions and emergency room visits and multivariable linear regression estimated adjusted developmental and parental quality of life scores with PPQ and PHQ-2 scores. The models were adjusted for confounders such as race/ethnicity, maternal education, primary language, neonatal co-morbidities, post discharge diagnoses and use of medical equipment. Beta coefficients (linear regression results) and odds ratios (ORs) with 95% confidence intervals (CIs) and two-sided *P*-values for individual variable categories are reported.

We also conducted an E-value analysis for the quality of life outcomes, which is a type of sensitivity analysis that quantifies unmeasured confounding to determine whether unmeasured confounding may have contributed to the observed effects [28]. As detailed in previous work conducted by our group [29], the E-value analysis addresses the extent to which unmeasured confounding may negate the observed results. A relatively low E-value in the context of statistical adjustments made suggests that the results could easily be nullified by a confounder. Conversely, a very high E-value relative to the point estimate may imply that the observed effect is in fact plausible, because the strength and association of the unmeasured confounder with the exposure group and outcome must be very high to negate the observed effect [29].

### Power calculation

A sample size of at least 150 with unequal groups achieves 99% power to reject the null hypothesis of equal means when the population difference in PPQ scores is 6 with a SD of 10 with a significance level (alpha) of 0.05 using a two sided two sample equal variance t-test (summary statement generated in PASS).

All statistical analyses were carried out using SAS, v. 9.4 (SAS Institute, Cary, NC, USA). E-values were then calculated using the R package “EValue” provided by the E-value creators [30].

## Results

**Fig. 1:**
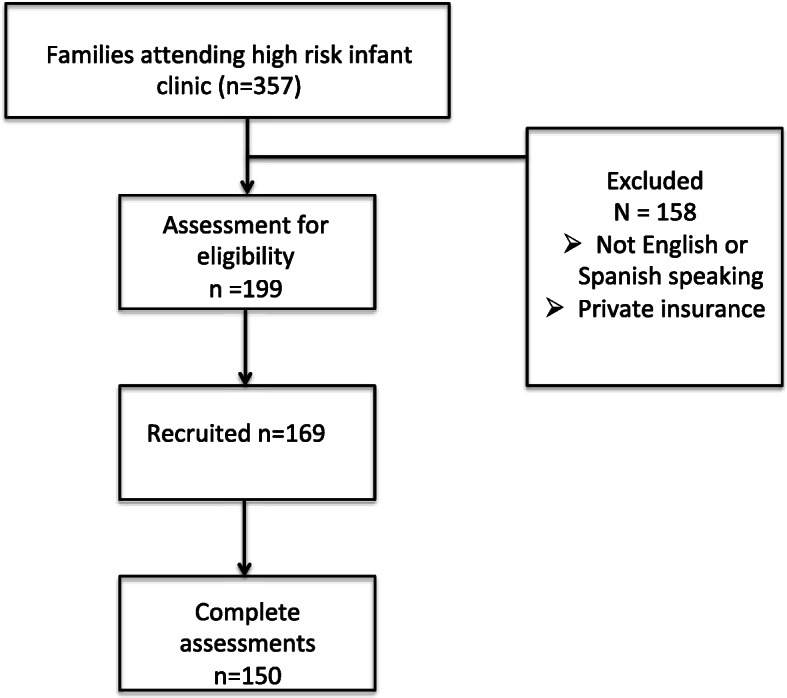
Enrollment and recruitment

One hundred sixty-nine participants were recruited and 150 completed PPQ and PHQ-2 screening (Fig. 1). The majority of our sample was Hispanic (68%), non-English speaking (67%) and reported an annual income of <$20,000 (58%). 34% of the participants had a positive PHQ-2 screen and 33% a positive PPQ screen (Table 1). Only 9 (< 7%) participants were fathers. Maternal education and language were not associated with PPQ or PHQ-2 scores. When looking at birth weight, gestational age, presence of neonatal comorbidities or use of equipment, there was no statistically significant differences between groups that screened positively and those who did not (Table 1).

**Table 1:** Socio-demographics and infant characteristics and caregiver mental health measure scores

	Perinatal Post-traumatic Stress Disorder Questionnaire (PPQ) (*N* = 139) N (%)				Patient Health Questionnaire-2 (PHQ-2) (*N* = 152) N (%)			
	Total	Positive	Negative	*P*-value	Total	Positive	Negative	*P*-value
Socio-demographics		46 (33)	93 (67)			51 (34)	99 (66)	
Person completing survey								
Mother	139	46 (100)	93 (100)	N/A	141	50 (98.0)	91 (91.9)	0.17
Father					9	1 (2.0)	8 (8.1)	
Race/ethnicity								
White non-Hispanic	10	1 (2.3)	8 (8.7)	0.32	10	2 (3.9)	8 (8.3)	0.07
Hispanic	102	35 (79.6)	67 (72.8)		109	41 (80.4)	68 (70.1)	
Black non-Hispanic	14	6 (13.6)	8 (8.7)		14	1 (2.0)	13 (13.4)	
Other	11	2 (4.6)	9 (9.8)		15	7 (13.7)	8 (8.3)	
Income ($/Year)								
Less than $20,000	82	32 (69.6)	50 (53.8)	0.34	88	36 (69.2)	52 (52.0)	0.35
$20,001–$40,000	31	9 (19.6)	22 (23.7)		35	9 (17.3)	26 (26.0)	
$40,001–$60,000	11	1 (2.2)	10 (10.8)		14	4 (7.7)	10 (10.0)	
$60,001–$80,000	8	2 (4.4)	6 (6.5)		7	2 (3.9)	5 (5.0)	
More than $80,000	7	2 (4.4)	5 (5.4)		8	1 (1.9)	7 (7.0)	
Highest level of education (either parent)								
≤ High school	45	13 (35.1)	32 (38.1)	0.75	48	19 (41.3)	29 (33.3)	0.36
At least some college	76	24 (64.9)	52 (61.9)		85	27 (58.7)	58 (66.7)	
Language								
Non-English	93	33 (71.7)	60 (64.5)	0.39	101	39 (76.5)	62 (62.6)	0.09
English	46	13 (28.3)	33 (35.5)		49	12 (23.5)	37 (37.4)	
Infant characteristics								
Birthweight (grams)								
< 500 to < 1000	58	19 (45.2)	39 (59.1)	0.53	62	21 (53.8)	41 (53.3)	0.78
≥ 1000 to < 1500	27	12 (28.6)	15 (22.7)		31	11 (28.2)	20 (26.0)	
≥ 1500 to < 2500	16	8 (19.1)	8 (12.1)		16	6 (15.4)	10 (13.0)	
≥ 2500	7	3 (7.1)	4 (6.1)		7	1 (2.6)	6 (7.7)	
Gestational age (weeks)								
< 24 to < 28	52	16 (34.8)	36 (51.4)	0.19	56	20 (48.9)	36 (43.4)	0.66
≥ 28 to < 32	45	21 (45.7)	24 (34.3)		48	17 (41.5)	31 (37.4)	
≥ 32 to < 34	12	7 (15.2)	5 (7.1)		13	3 (7.3)	10 (12.1)	
≥ 34 to <37	7	2 (4.4)	5 (7.1)		7	1 (2.4)	6 (7.2)	
Neonatal co-morbidities^a^								
Yes	94	36 (78.3)	58 (62.4)	0.06	102	35 (67.3)	67 (67.0)	0.97
No	45	10 (21.7)	35 (37.6)		50	17 (32.7)	33 (33.0)	
Use of medical equipment^b^								
Yes	43	15 (32.6)	28 (30.1)	0.76	46	12 (23.1)	34 (34.0)	0.16
No	96	31 (67.4)	65 (69.9)		106	40 (76.9)	66 (66.0)	
≥ 2 clinic appointments/month								
Yes	102	34 (73.9)	68 (73.1)	0.92	115	37 (71.2)	78 (78.0)	0.35
No	37	12 (26.1)	25 (26.9)		37	15 (28.8)	22 (22.0)	
Post discharge diagnoses^c^								
Yes	100	37 (80.4)	63 (90.0)	0.14	107	37 (90.2)	70 (84.3)	0.42
No	16	9 (19.6)	7 (10.0)		17	4 (9.8)	13 (15.7)	

Characteristics of neonates are shown as mean (standard deviation) for PPQ-14 and N (%) for PHQ-2 scores. P-values derived using t-test (for 2 group comparison) and ANOVA test (for multi-group comparison) for continuous variables, and chi-square and fisher’s exact test for categorical variables

^a^Neonatal co-morbidities include at least one diagnosis of: fetal growth restriction, surfactant deficiency, necrotizing enterocolitis, intraventricular hemorrhage grade 3 or 4, patent ductus arteriosus, retinopathy of prematurity

^b^Use of medical equipment includes: oxygen, tracheostomy, wheelchair, adaptive stroller, feeding tube

^c^Post discharge diagnoses include at least one diagnosis of: attention deficit hyperactivity disorder, autism, global developmental delay, cerebral palsy

**Fig. 2:**
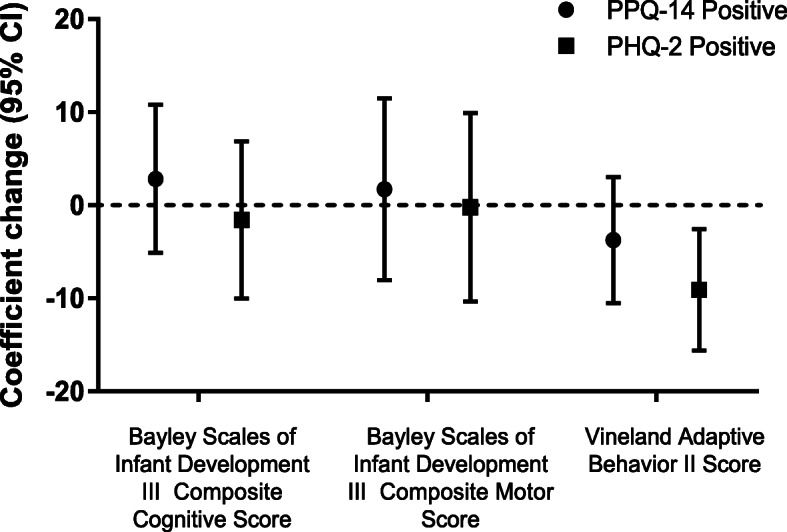
Adjusted association of parents with positive post-traumatic stress and depression screening and child developmental outcomes (*n* = 89)

As demonstrated in Fig. 2, after adjusting for confounders, we identified that a positive PHQ-2 depression score was associated with a negative unit (95% CI) change on the infant’s Vineland score of − 9.08 (− 15.6, − 2.6) (*p* < 0.01) in 89 participants. There were no significant association between participant depression and stress scores and infant Bayley-III scores (motor or cognitive). As anticipated, use of medical equipment was associated (95% CI) with lower Bayley-III motor and cognitive scores and VABS-II scores (Supplemental Tables 1 and 2) independent of PPQ screening: − 18.7 (− 27, − 9.8) and − 24.3 (− 35, − 13) and − 11.3 (− 19, − 4). Similarly, Bayley-III motor and cognitive scores (95% CI) and VABS-II scores were lower independent of PHQ-2 screening, − 18.6 (− 28, − 9.7) and − 24 (− 35, − 13) and − 12 (− 19, − 5). Additionally, while the sample was small, Black Non-Hispanic race was also associated (95% CI) with lower Bayley-III motor scores, − 41 (− 66, − 15) independent of PPQ screening, and independent of PHQ-2 screening, − 42 (− 67, − 17) (Supplemental Table 1 and 2). After adjusting for observable confounders, there was no statistically significant association between PPQ or PHQ-2 screening and rehospitalizations and emergency room visits (Table 2).

**Table 2:** Adjusted association of maternal mental health measure scores and medical outcomes (re-hospitalization or emergency room visit)

	OR (95% CI)	*P*-value		OR (95%CI)	*P*-value
Positive Perinatal Post-traumatic Stress Disorder Questionnaire (PPQ) screen	1.29 (0.5, 3.29)	0.6	Positive Patient Health Questionnaire- 2 Screen	1.06 (0.42, 6.69)	0.89
Race					
Non-Hispanic	Reference			Ref	
Hispanic	1.17 (0.34, 3.98)	0.8		1.16 (0.34, 3.96)	0.82
Maternal Education					
≤ High School	Reference			Ref	
Some college	1.46 (0.53, 3.98)	0.46		1.44 (0.52, 3.96)	0.48
Primary language					
English	Reference			Ref	
Non-English	0.64 (0.21, 1.99)	0.44		0.61 (0.2, 1.88)	0.39
Annual household income					
Less than $20,000	1.6 (0.54, 4.71)	0.52		1.57 (0.52, 4.68)	0.56
$20,001–$40,000	Reference			Ref	
$40,001–$60,000	1.67 (0.23, 12)	0.66		1.72 (0.24, 12.31)	0.64
More than $60,000	0.8 (0.09, 6.96)	0.62		0.81 (0.09, 7.04)	0.62
Infant chronologic age (month)	1.11 (1.04, 1.9)	0.003		1.12 (1.04, 1.20)	0.003
Medical equipment^a^	3.47 (1.24, 9.76)	0.02		3.48 (1.24, 9.74)	0.02

Adjusted odds ratios with 95% confidence intervals are shown vs. reference categories unless otherwise noted. Model adjusted for race/ethnicity, maternal education, language, annual household income, birth weight, use of medical equipment, and enrollment in early intervention

^a^Use of medical equipment includes: oxygen, tracheostomy, wheelchair, adaptive stroller, feeding tube

**Fig. 3:**
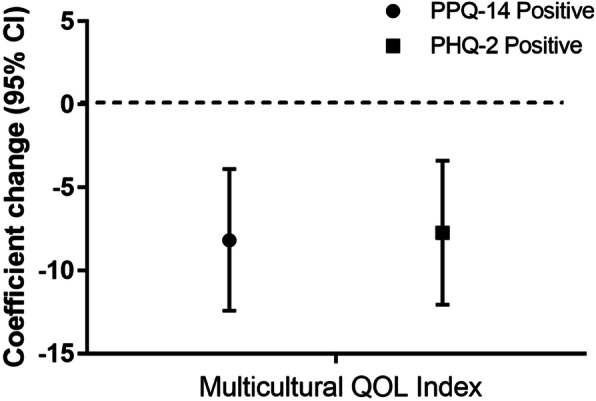
Quality of life scores and positive post-traumatic stress and depression screening

Positive PPQ and PHQ-2 screening scores were associated with a negative unit (95% CI) unit change on the participant Multicultural Quality of Life Index score of − 8.1 (− 12, − 3.9)(*p* < 0.01) and − 7.7 (− 12, − 3) (*p* = 0.01) respectively (Fig. 3 and Supplemental Table 3). A subsequent E-value analysis demonstrated that the associated E-value (95% CI) for PPQ was 2.27 (1.03) and for the PHQ-2 screen was 2.21 (1.62).

## Discussion

More than one-third of the participants in this sample screened positively for PTSD and depression. Screening scores positive for stress and depression were associated with a negative change in some infant development scores and maternal quality of life scores. While studies have examined the impact of maternal mental health on the mother and fetus [31], few have examined the prevalence of PTSD and depression after NICU discharge in a diverse and underserved population as presented [32].

Studies have found that Hispanic and Black mothers have higher rates of post partum depression due to lack of social support, access, trust, past depression and other factors and Black women may even be less likely to seek treatment due to stigma [33]. Moreover, studies have found that among Hispanic women, acculturation was associated with higher rates of perinatal depression suggesting nativity may affect outcomes [34]. Previous maternal mental health disease has also been associated with lower levels of readiness for discharge [35]. In our sample, one third of participants screened positively for depression which is consistent with previous literature [36, 37]. Similarly, more than a third of participants screened positively for post-traumatic stress. Previous work has identified that maternal distress is often marked by post-traumatic stress, depression and anxiety and prevalent in mothers whose infants have been hospitalized in the NICU [38]. Understanding both the mother’s personal history of mental health disorders, social complexity and support systems are important when interpreting depression and post-traumatic stress screening [39].

We identified a negative unit change in our VABS-II score among Spanish speaking families. Previous work has found that children of US-born Latinas with depression have poorer developmental outcomes than foreign-born Latinas [40]. Social capital has been found to improve maternal health of foreign-born Latina women [41]. Moreover, there is evidence of Latina paradox in some situations, where babies born to US-born Latina women face similar outcomes to Whites while babies of foreign-born Latinas have better outcomes in terms of prematurity and birthweight [42]. Similarly, it has been shown that among families with limited English proficiency, there were higher incidence rates of completion of influenza vaccines and preventive visits [43]. This has been attributed to concepts like “*simpatia* (politeness to avoid conflict)” or “*respeto* (respect)” as well as “marianismo” or female gender role in Hispanic culture. Moreover, it has been demonstrated that poverty, toxic stress and preterm birth can also impact developmental outcomes [44]. Also, while not appropriately powered, we identified that Black race was associated with worse neurodevelopmental outcomes independent of parental stress or depression. A recent publication by the Eunice Kennedy Shriver National Institute of Child Health and Human Development Neonatal Research Network found that neurodevelopmental impairment has increased across all ethnic groups and not just minorities (increase from 2006 to 2014: black infants, 70%; Hispanic infants, 123%; white infants, 130%) [45]. Studies have demonstrated that concepts like structural racism and discrimination are associated with maternal health outcomes [46–49] and future work should be conducted to demonstrate how they might impact our findings.

We did find a significant association between screening positive for post-traumatic stress and depression symptoms and lower quality of life scores. Previous studies have established how the act of caring for a preterm infant negatively affects family dynamics and maternal quality of life [5] but may improve over time [50]. In this study we used one of the same scales previously used to assess quality of life the Multicultural Quality of Life Index (MQLI) which assessed a mothers’ overall health and well being including physical well being and social emotional support. Showing how maternal post-traumatic stress and depression symptoms affect quality of life establishes an important potential modifiable risk factor. We also conducted a sensitivity analysis with our E-value analysis and found that that a moderate confounder may affect our results. For example, for participants who screened positive for post-traumatic stress (PPQ), the associated E-value (95% CI) was 2.27 (1.03) and for the PHQ-2 screen was 2.21 (1.62). Constructs like social support, living situation, nutrition and previous mental health disease and coping may also contribute to findings. Further evaluation is thus warranted on the possible protective effects increased social support and improved quality of life have on minimizing poor postpartum mental health and child health outcomes in preterm infants. This study reinforces the need to universally screen NICU mothers for PTSD and depression.

## Limitations

This was a cross-sectional study; thus directionality of neither association nor causality is able to be determined. The tools used to assess post-traumatic stress and depression were screening tools and not diagnostic ones. Additionally, despite the strong association between Black race and worse outcomes on cognitive, motor and adaptive developmental screens seen in the adjusted logistic regression models, the sample size of Black mothers in the study was very small and was not powered to evaluate the association of race with developmental and adaptive behavioral outcomes. Moreover, given this study was done as a survey requiring literacy, those with literacy challenges who may represent a population with even greater socioeconomic barriers differing mental health outcomes, are not adequately captured in this study. Also, the power calculation was performed to detect the difference in PPQ scores and the secondary outcomes (infant and neurodevelopmental outcomes) were not powered.

## Future directions

This study reinforces the need to universally screen NICU mothers for PTSD and depression. Additionally, dedicated evaluation on the association of race and cognitive and adaptive behavioral outcomes could help identifying other vulnerable populations who could potentially benefit from targeting resources and interventions. Specific parental treatment interventions have been shown to reduce trauma symptoms and depression [51, 52]. A recent meta-analysis has also demonstrated that cognitive behavioral therapy (CBT) is most effective to reduce depressive symptoms [53]. Telehealth and internet-based mental health services may also be promising solution for low-SES mothers and high-risk mothers (e.g., those in Neonatal Intensive Care Units) to access mental health services. Hynan et al. review steps to include telehealth services in providing psychosocial support to families, including using telemedicine for screening, maintaining compliance with the American Telemedicine Association and American Psychological Association, and for staff to familiarize themselves with web-based support sites [54, 55]. Several NICUs already employ web-based cameras, Skype, and FaceTime to allow parents to check on their hospitalized children. Augmenting these virtual connections might be a natural next step.

## Conclusions

Screening scores positive for stress and depression symptoms were associated with a negative change in some infant development scores and maternal quality of life scores. Thoughtful screening programs for maternal stress and depression symptoms should be instituted.

## Supplementary Information

**Additional file 1: Supplemental Table 1.** Association of maternal mental health measure scores and neurodevelopmental scores (*n* = 89). **Supplemental Table 2.** Association of maternal mental health measure scores and neurodevelopmental scores (*n* = 89). **Supplemental Table 3.** Association of maternal mental health measure scores and quality of life measures.

## Data Availability

The datasets used and/or analyzed during the current study available from the corresponding author on reasonable request.

## Abbreviations

NICU: Neonatal Intensive Care Unit
PTS: Post traumatic stress
PTSD: Post traumatic stress disorder
