# Supplementary material for: Maternal post-traumatic stress and depression symptoms and outcomes after NICU discharge in a low-income sample: a cross-sectional study
Source: BMC Pregnancy Childbirth. 2021 Jan 12;21:48. doi: 10.1186/s12884-020-03536-0 (PMC7802207; doi:10.1186/s12884-020-03536-0)
Supplement: Supplementary file 1 — Additional file 1: Supplemental Table 1. Association of maternal mental health measure scores and neurodevelopmental scores (n = 89). Supplemental Table 2. Association of maternal mental health measure scores and neurodevelopmental scores (n = 89). Supplemental Table 3. Association of maternal mental health measure scores and quality of life measures. [file 12884_2020_3536_MOESM1_ESM.docx]

Supplemental Materials

**Supplemental Table 1. Association of maternal mental health measure scores and neurodevelopmental scores (n=89)**

|  | **Bayley Scales of Infant Development III Composite Cognitive Score** | | | **Bayley Scales of Infant Development III Composite Motor Score** | | | **Vineland Adaptive Behavior II Score** | | |
| --- | --- | --- | --- | --- | --- | --- | --- | --- | --- |
|  | Beta  Coefficient | 95% Confidence Interval | P value | Beta  Coefficient | 95% Confidence Interval | P value | Beta  Coefficient | 95% Confidence Interval | P value |
| **Unadjusted Model** |  |  |  |  |  |  |  |  |  |
| PPQ-14 Positive | 0.76 | -7.61, 9.14 | 0.86 | 0.92 | -9.19, 11.03 | 0.86 | -3.60 | -10.28, 3.08 | 0.29 |
| **Adjusted Model** |  |  |  |  |  |  |  |  |  |
| PPQ-14 Positive | 2.83 | -5.12, 10.79 | 0.48 | 1.72 | -8.05, 11.49 | 0.73 | -3.76 | -10.53, 3.01 | 0.27 |
| Race |  |  |  |  |  |  |  |  |  |
| White Non-Hispanic | Ref |  |  | Ref |  |  | Ref |  |  |
| Black Non-Hispanic | -40.98 | -66.07, -15.89 | 0.002 | -23.72 | -54.21, 6.79 | 0.12 | -12.68 | -33.3, 7.97 | 0.22 |
| Hispanic | -8.57 | -28.72, 11.58 | 0.40 | -18.98 | -43.46, 5.50 | 0.13 | -4.55 | -20.92, 11.83 | 0.58 |
| Other | -7.38 | -37.01, 22.24 | 0.62 | -14.20 | -50.21, 21.80 | 0.43 | 5.87 | -18.25, 29.99 | 0.63 |
| Maternal Education |  |  |  |  |  |  |  |  |  |
| ≤ High School | Ref |  |  | Ref |  |  | Ref |  |  |
| Some college | -1.51 | -10.06, 7.05 | 0.73 | -8.30 | -18.62, 2.01 | 0.11 | -8.59 | -15.63, -1.55 | 0.02 |
| Primary language |  |  |  |  |  |  |  |  |  |
| English | Ref |  |  | Ref |  |  | Ref |  |  |
| Non-English | -9.02 | -20.19, 2.15 | 0.11 | -4.22 | -17.83, 9.38 | 0.54 | -7.96 | -17.24, 1.33 | 0.09 |
| Annual household income |  |  |  |  |  |  |  |  |  |
| Less than $20,000 | 5.81 | -3.79, 15.40 | 0.23 | 3.24 | -8.43, 14.91 | 0.58 | 2.78 | -5.37, 10.93 | 0.50 |
| $20,001-$40,000 | Ref |  |  | Ref |  |  | Ref |  |  |
| $40,001-$60,000 | 23.64 | 2.85, 44.43 | 0.03 | 23.31 | -1.98, 48.60 | 0.07 | 5.17 | -12.11, 22.44 | 0.55 |
| $60,001-$80,000 | -26.73 | -53.45, -0.01 | 0.05 | -12.93 | -45.35, 19.48 | 0.43 | -1.35 | -23.38, 20.69 | 0.90 |
| More than $80,000 | -14.14 | -49.64, 21.35 | 0.43 | 2.24 | -40.86, 45.34 | 0.92 | -9.00 | -38.51, 20.49 | 0.54 |
| Infant chronologic age (month) | -0.81 | -1.50, -0.12 | 0.03 | -0.46 | -1.27, 0.36 | 0.27 | -0.54 | -1.15, 0.06 | 0.08 |
| Birthweight (grams) | 0.002 | -0.004, 0.009 | 0.48 | 0.003 | -0.005, 0.01 | 0.44 | 0.002 | -0.006, 0.009 | 0.66 |
| Medical equipment^a^ | -18.74 | -27.65, -9.84 | <0.001 | -24.26 | -35.08, -13.44 | <0.001 | -11.37 | -18.94, -3.79 | 0.004 |
| Early Intervention | -2.51 | -10.72, 5.70 | 0.54 | -3.80 | -13.76, 6.16 | 0.45 | -0.54 | -7.23, 6.16 | 0.87 |

Estimated beta coefficient with 95% confidence intervals are shown vs. reference categories unless otherwise noted. Adjusted model controlled for race/ethnicity, maternal education, language, annual household income, birth weight, use of medical equipment, and enrollment in early intervention.

^a^Use of medical equipment includes: oxygen, tracheostomy, wheelchair, adaptive stroller, feeding tube

**Supplemental Table 2: Association of maternal mental health measure scores and neurodevelopmental scores (n=89)**

|  | **Bayley Scales of Infant Development III Composite Cognitive Score** | | | **Bayley Scales of Infant Development III Composite Motor Score** | | | **Vineland Adaptive Behavior II Score** | | |
| --- | --- | --- | --- | --- | --- | --- | --- | --- | --- |
|  | Beta  Coefficient | 95% Confidence Interval | P value | Beta  Coefficient | 95% Confidence Interval | P value | Beta  Coefficient | 95% Confidence Interval | P value |
| **Unadjusted Model** |  |  |  |  |  |  |  |  |  |
| PHQ-2 positive | 0.82 | -8.35, 9.98 | 0.86 | -0.28 | -11.06, 10.50 | 0.96 | -5.49 | -12.87, 1.89 | 0.14 |
| **Adjusted Model** |  |  |  |  |  |  |  |  |  |
| PHQ-2 positive | -1.59 | -10.03, 6.84 | 0.71 | -0.21 | -10.35, 9.93 | 0.97 | -9.08 | -15.61, -2.55 | <0.01 |
| Race |  |  |  |  |  |  |  |  |  |
| White Non-Hispanic | Ref |  |  | Ref |  |  | Ref |  |  |
| Black Non-Hispanic | -42.05 | -67.10, -17.01 | 0.001 | -24.31 | -54.67, 6.06 | 0.11 | -12.05 | -31.46, 7.36 | 0.22 |
| Hispanic | -9.08 | -29.25, 11.08 | 0.37 | -19.31 | -43.74, 5.13 | 0.12 | -3.91 | -19.30, 11.47 | 0.61 |
| Other | --8.24 | -37.90, 21.41 | 0.58 | -14.66 | -50.62, 21.30 | 0.42 | 6.42 | -16.26, 29.11 | 0.57 |
| Maternal Education |  |  |  |  |  |  |  |  |  |
| ≤ High School | Ref |  |  | Ref |  |  | Ref |  |  |
| Some college | -1.30 | -10.06, 7.47 | 0.77 | -8.34 | -18.85, 2.17 | 0.12 | -6.82 | -13.48, -0.16 | 0.05 |
| Primary language |  |  |  |  |  |  |  |  |  |
| English | Ref |  |  | Ref |  |  | Ref |  |  |
| Non-English | -9.14 | -20.43, 2.15 | 0.11 | -4.40 | -18.20, 9.40 | 0.53 | -5.38 | -14.21, 3.45 | 0.23 |
| Annual household income |  |  |  |  |  |  |  |  |  |
| Less than $20,000 | 5.91 | -3.89, 15.69 | 0.23 | 3.11 | -8.78, 15.00 | 0.60 | 5.62 | -2.27, 13.50 | 0.16 |
| $20,001-$40,000 | Ref |  |  | Ref |  |  | Ref |  |  |
| $40,001-$60,000 | 23.05 | 2.12, 43.99 | 0.03 | 23.09 | -2.31, 48.50 | 0.07 | 3.69 | -12.62, 19.99 | 0.65 |
| $60,001-$80,000 | -42.05 | -67.09, -17.01 | 0.001 | -12.64 | -45.47, 20.19 | 0.44 | 2.78 | -18.23, 23.79 | 0.79 |
| More than $80,000 | -8.24 | -37.89, 21.41 | 0.58 | 2.71 | -40.38, 45.80 | 0.90 | -11.39 | -39.18, 16.41 | 0.41 |
| Infant chronologic age (month) | -0.83 | -1.52, -0.14 | 0.02 | -0.45 | -1.27, 0.36 | 0.27 | -0.56 | -1.13, 0.01 | 0.06 |
| Birthweight (grams) | 0.002 | -0.004, 0.009 | 0.48 | 0.003 | -0.005, 0.01 | 0.43 | 0.003 | -0.003, 0.009 | 0.29 |
| Medical equipment^a^ | -18.62 | -27.56, -9.69 | <0.01 | -24.16 | -35.01, -13.31 | <0.01 | -12.27 | -19.31, -5.23 | <0.01 |
| Early Intervention | -2.23 | -10.49, 6.03 | 0.59 | -3.61 | -13.62, 6.40 | 0.47 | 0.20 | -6.12, 6.52 | 0.95 |

Estimated beta coefficient with 95% confidence intervals are shown vs. reference categories unless otherwise noted. Adjusted model controlled for race/ethnicity, maternal education, language, annual household income, birth weight, use of medical equipment, and enrollment in early intervention.

^a^Use of medical equipment includes: oxygen, tracheostomy, wheelchair, adaptive stroller, feeding tube

**Supplemental Table 3: Association of maternal mental health measure scores and quality of life measures**

|  | **Multicultural QOL Index** | | |
| --- | --- | --- | --- |
|  | β | 95% CI | P value |
| **Unadjusted Model** |  |  |  |
| PPQ-14 positive | -13.38 | -18.98, -7.77 | <0.01 |
| **Adjusted Model** |  |  |  |
| PPQ-14 positive | -8.17 | -12.43, -3.90 | <0.01 |
| Race |  |  |  |
| White Non-Hispanic | Ref |  |  |
| Black Non-Hispanic | -2.36 | -14.94, 10.21 | 0.71 |
| Hispanic | -2.52 | -13.71, 8.67 | 0.66 |
| Other | -10.42 | -25.61, 4.78 | 0.18 |
| Maternal Education |  |  |  |
| ≤ High School | Ref |  |  |
| Some college | -1.38 | -5.99, 3.23 | 0.55 |
| Primary language |  |  |  |
| English | Ref |  |  |
| Non-English | -0.53 | -5.72, 4.67 | 0.84 |
| Annual household income |  |  |  |
| Less than $20,000 | -2.54 | -7.34, 2.25 | 0.29 |
| $20,001-$40,000 | Ref |  |  |
| $40,001-$60,000 | -0.43 | -9.64, 8.79 | 0.93 |
| $60,001-$80,000 | -15.64 | -31.39, 0.11 | 0.05 |
| More than $80,000 | 0.09 | -12.47, 12.66 | 0.99 |
| Infant chronologic age (month) | 0.12 | -0.18, 0.41 | 0.43 |
| Birthweight (grams) | 0.001 | -0.18, 0.41 | 0.43 |
| Medical equipment^a^ | 0.65 | -3.88, 5.18 | 0.78 |
| Early Intervention | -2.67 | -7.05, 1.71 | 0.23 |

Estimated beta coefficient with 95% confidence intervals are shown vs. reference categories unless otherwise noted. Adjusted model controlled for race/ethnicity, maternal education, language, annual household income, birth weight, use of medical equipment, and enrollment in early intervention.

^a^Use of medical equipment includes: oxygen, tracheostomy, wheelchair, adaptive stroller, feeding tube
